# High-Frequency, Functional HIV-Specific T-Follicular Helper and Regulatory Cells Are Present Within Germinal Centers in Children but Not Adults

**DOI:** 10.3389/fimmu.2018.01975

**Published:** 2018-09-12

**Authors:** Julia Roider, Takashi Maehara, Abigail Ngoepe, Duran Ramsuran, Maximilian Muenchhoff, Emily Adland, Toby Aicher, Samuel W. Kazer, Pieter Jooste, Farina Karim, Warren Kuhn, Alex K. Shalek, Thumbi Ndung'u, Lynn Morris, Penny L. Moore, Shiv Pillai, Henrik Kløverpris, Philip Goulder, Alasdair Leslie

**Affiliations:** ^1^Africa Health Research Institute, University of KwaZulu-Natal, Durban, South Africa; ^2^Department of Paediatrics, Peter Medawar Building for Pathogen Research, Oxford University, Oxford, United Kingdom; ^3^HIV Pathogenesis Programme, Doris Duke Medical Research Institute, University of KwaZulu-Natal, Durban, South Africa; ^4^Department of Infectious Diseases, Medizinische Klinik IV, Ludwig-Maximilians-University Munich, Munich, Germany; ^5^Ragon Institute of Massachusetts General Hospital, Massachusetts Institute of Technology and Harvard University, Cambridge, MA, United States; ^6^Department of Virology, Max von Pettenkofer Institute, Ludwig-Maximilians-University Munich, Munich, Germany; ^7^Partner Site Munich, German Center for Infection Research, Munich, Germany; ^8^Department of Chemistry and Institute for Medical Engineering and Science, Massachusetts Institute of Technology, Cambridge, MA, United States; ^9^Broad Institute of MIT and Harvard, Cambridge, MA, United States; ^10^Paediatric Department, Kimberley Hospital, Kimberley, South Africa; ^11^Department of Otorhinolaryngology, Stanger Hospital, KwaZulu-Natal, South Africa; ^12^Max Planck Institute for Infection Biology, Berlin, Germany; ^13^Department of Infection and Immunity, University College London, London, United Kingdom; ^14^Centre for HIV and STIs, National Institute for Communicable Diseases of the National Health Laboratory Service, Johannesburg, South Africa; ^15^Faculty of Health Sciences, University of the Witwatersrand, Johannesburg, South Africa; ^16^Center for the AIDS Programme of Research in South Africa, Durban, South Africa; ^17^Department of Immunology and Microbiology, University of Copenhagen, Copenhagen, Denmark

**Keywords:** pediatric HIV infection, broadly neutralizing antibodies (bnAb), T-follicular helper cells (Tfh), T-follicular regulatory helper cells (Tfreg), follicular CD8 T-cells, germinal center, vaccination

## Abstract

Broadly neutralizing antibodies (bnAbs) against HIV-1 are an effective means of preventing transmission. To better understand the mechanisms by which HIV-specific bnAbs naturally develop, we investigated blood and lymphoid tissue in pediatric infection, since potent bnAbs develop with greater frequency in children than adults. As in adults, the frequency of circulating effector T-follicular helper cells (T_FH_) in HIV infected, treatment naïve children correlates with neutralization breadth. However, major differences between children and adults were also observed both in circulation, and in a small number of tonsil samples. In children, T_FH_ cells are significantly more abundant, both in blood and in lymphoid tissue germinal centers, than in adults. Second, HIV-specific T_FH_ cells are more frequent in pediatric than in adult lymphoid tissue and secrete the signature cytokine IL-21, which HIV-infected adults do not. Third, the enrichment of IL-21-secreting HIV-specific T_FH_ in pediatric lymphoid tissue is accompanied by increased T_FH_ regulation via more abundant regulatory follicular T-cells and HIV-specific CXCR5+ CD8 T-cells compared to adults. The relationship between regulation and neutralization breadth is also observed in the pediatric PBMC samples and correlates with neutralization breadth. Matching neutralization data from lymphoid tissue samples is not available. However, the distinction between infected children and adults in the magnitude, quality and regulation of HIV-specific T_FH_ responses is consistent with the superior ability of children to develop high-frequency, potent bnAbs. These findings suggest the possibility that the optimal timing for next generation vaccine strategies designed to induce high-frequency, potent bnAbs to prevent HIV infection in adults would be in childhood.

## Introduction

A protective HIV-1 vaccine is likely to require the generation of high affinity antibodies recognizing most of the circulating HIV strains worldwide (1–4). Broadly neutralizing antibodies (bnAbs) can prevent SIV/SHIV infection in non-human primates (5–9). However, the mechanism by which a vaccine might elicit bnAbs against HIV remains unclear (10–12).

Broadly neutralizing antibodies are observed in approximately 20% of HIV-infected adults (13–15) and often take years to develop (16). By contrast, bnAbs develop in 75–89% of HIV-infected children, are substantially more potent than those observed in adults (17), and develop as early as the first year of life (18). In a direct comparison between infant and adult responses to the same gp120 vaccine, HIV-uninfected children made higher-magnitude antibody responses (19, 20), further suggesting that children are generally better at generating antibodies than adults. As in adults (21), the generation of bnAbs in infected children is related to viral load (17, 18). However, viral load is only weakly correlated with neutralization breadth (17) indicating that other factors play a more important role.

T-follicular helper (T_FH_) cells within germinal centers (GC) are CD4 T-cells that provide the B- cell help required to produce increasingly high affinity antibodies over the course of an infection, by the process of somatic hypermutation. Many bnAbs identified to date show a high degree of somatic hypermutation (1, 3), suggesting an efficient GC response mediated by T_FH_ cells (4, 22, 23). However, as B-cell affinity maturation relies on competition between B-cell clones, the help provided by T_FH_ needs to be a limiting resource for effective selection to occur (24, 25). Thus, regulation of T_FH_ in the GC by T-follicular regulatory CD4 cells (T_FR_) is another critical determinant of antibody breadth and potency in HIV infection. T_FR_ are derived from thymic T_REG_ precursors, express FoxP3, CD25, and low levels of CD127, and subsequently acquire T_FH_ markers (PD1, CXCR5 and Bcl-6) (26–28). These T_FR_ cells contribute to the regulation and proliferation of T_FH_ and GC B-cells (29, 30), and skewing of T_FR_/T_FH_ ratio leads to unchecked expansion of T_FH_ and an ineffective humoral immune response (31, 32). Growing evidence from murine models show that T_FR_ cells are necessary to ensure the quality of the antibody response (33, 34). CXCR5+ve CD8 T-cells in secondary lymphoid tissue and in circulation can also regulate the T_FH_ response (35–37). These CXCR5+ve CD8 T-cells reduce GC responses through perforin-dependent lysis of GC T_FH_ cells and prevent autoantibody development in murine and SIV models (38–40).

Understanding the immune environment in which high-affinity antibodies develop is a critical step toward new vaccination strategies. We therefore studied the T_FH_ response and its regulatory counterparts in HIV-infected children and adults to understand the immune conditions that do and do not, respectively, support HIV bnAb development.

## Materials and methods

### Study participants

Peripheral blood mononuclear cells (PBMC) of 38 vertically HIV-1 C clade-infected ART-naïve children with matched neutralization data (17) were studied. Additional pediatric and adult samples were obtained from clinics in Durban, South Africa (Ithembalabantu Clinic and Prince Mshiyeni Hospital). Tonsil specimens were obtained from medically indicated tonsillectomies carried out in KwaZulu-Natal (Stanger Hospital in Stanger, Addington Hospital in Durban). All tonsillectomies in this study were conducted for either chronic obstructive symptoms (e.g., snoring) due to tonsillar hypertrophy and/or recurrent tonsillitis. All procedures were elective and, if conducted for recurrent tonsillitis, were carried out after infection and associated inflammation had resolved, as determined by the clinician and typically 6 weeks or more after the last episode. The clinical characteristics of the study cohort are shown in Table S1. Adult participants on whom neutralization assays were undertaken are from the South African CAPRISA 002 cohort, as previously described (41). Viral load measurements were performed as described previously (17). Informed consent was obtained from all adult study participants or from the caregivers of pediatric participants where appropriate. Additionally, assent to participate in the study was given directly by children from the age of six and above. Studies were approved by the University of the Free State Ethics Committee, Bloemfontein; Biomedical Research Ethics Committee, University of KwaZulu-Natal, Durban; and Research Ethics Committee, University of Oxford.

### Sample processing–PBMC and tonsils

PBMCs were isolated by Ficoll density gradient centrifugation and stored in liquid nitrogen until use. Tonsil mononuclear cells (TMCs) were obtained by mechanical and enzymatic disaggregation using the gentleMACS system (Miltenyi). Mononuclear cells were then isolated by Ficoll density gradient centrifugation and used directly or preserved as above.

### Virus neutralization assays

The ability of plasma from infected children and adults to neutralize HIV was measured as described before (17).

### Flow cytometry and ICS assays

PBMCs and TMCs were stained with fluorescent monoclonal antibodies against markers previously associated with T_FH_ cells Table S2. Briefly, cells were thawed and rested in R10 medium for 3 h at 37°C in 5% CO_2_ and either directly stained with the phenotypic panel as described below or used for Intracellular Cytokine Staining (ICS) by stimulating with SEB at 1 μg/ml or with pools of overlapping 18-mer HIV peptides (Gag and Env at 2 μg/ml for each peptide) in the presence of anti-CD28 and anti-CD49 at 1 μg/ml (BD Biosciences). After 1 h of incubation at 37°C, Brefeldin A and Monensin (5 μg/ml; BD biosciences) were added and the cells were incubated overnight (14 h), washed and stained in the dark for 20 min with antibody cocktail and live dead stain (Fixable Blue, Thermo) and fixed. ICS was performed by standard methods (42), using fix/perm solution (BD), 20% Goat serum for Fc-receptor blockade and antibodies listed as above. Rainbow beads were run at every experiment to ensure interexperimental consistency. Flow cytometry acquisition was performed on a BD LSRFortessa within 5 h of staining and analyzed using FlowJo version 9.9.5.

### Multi-color immunofluorescence staining

Tissue samples were fixed in formalin, embedded in paraffin, and sectioned. These specimens were incubated with antibodies: anti-CD4 (clone: CM153A; Biocare), CD8 (clone: ab85792; Abcam), CXCR5 (clone: MAB190; R&D) and Foxp3 (clone: ab22510; Abcam) followed by incubation with secondary antibody using a SuperPicTure™ Polymer Detection Kit (Invitrogen) and an Opal™ 3-Plex Kit (Fluorescein, Cyanine3, and Cyanine5). The samples were mounted with ProLong^TM^ Gold Antifade mountant containing DAPI (Invitrogen).

### Statistical analyses

Statistical analyses were undertaken using Prism GraphPad Software version 7.0; for comparisons between two groups Mann–Whitney–Wilcoxon test was applied and for comparisons >2 groups Kruskal-Wallis test with Benjamini, Krieger and Yekutieli's correction for multiple comparisons. All correlations were performed using the Spearman rank method with exact permutation *P*-values calculated. All *P*-values are two-sided with a cut off of *p* > 0.05. For **Figure 5**, the Spice (Simplified Presentation of Incredibly Complex Evaluations) package was used to calculate permutation *P*-values between Spice charts (43).

## Results

### Circulating effector memory T_FH_ cells are abundant in HIV-infected children and correlate with neutralization breadth

**Figure 1:**
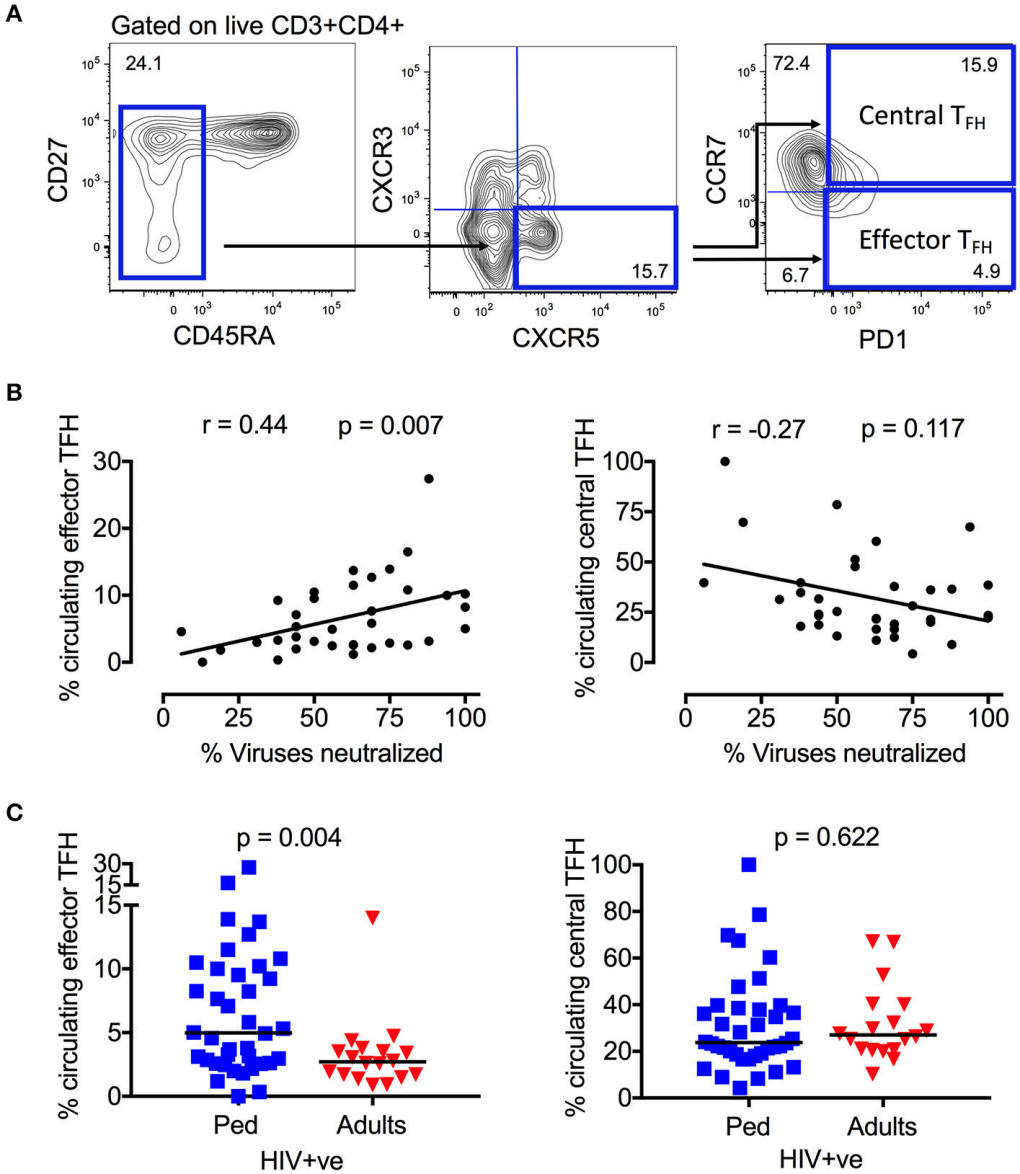
Circulating effector memory T_FH_ cells correlate with neutralization breadth in HIV infected children. **(A)** Gating strategy of CD4^+^CD45RA^−^CXCR5^+^CXCR3^−^PD1^+^ T_FH_ cells in peripheral blood. The majority of circulating T_FH_ cells express a resting central memory phenotype (CCR7+) in contrast to the effector memory (CCR7–) phenotype. **(B)** Positive correlation between circulating effector T_FH_ cells and neutralization breadth (*r* = 0.44, *p* = 0.007; left) and inverse correlation between circulating central T_FH_ cells and neutralization breadth in HIV infected children (*n* = 36) (*r* = −0.27, *p* = 0.117; right). Calculations were made by Spearman's rank correlation test. **(C)** Increased frequency of circulating effector T_FH_ cells in HIV-infected, ART-naïve children (blue squares; *n* = 38) compared to infected adults (red triangles; *n* = 18) (*p* = 0.004; left). No significant differences in frequency of circulating central T_FH_ between the groups (n.s., right). Comparisons between >2 groups were calculated using Kruskal-Wallis test and corrected for multiple comparisons. In scatter plots medians are shown.

Neutralizing antibody breadth was previously determined in plasma samples from 85 ART-naïve, HIV-infected children (17). Although 75% of pediatric samples were broadly neutralizing (i.e., neutralized >50% of the virus panel tested), neutralization breadth ranged from 0 to 100%. To investigate the T-cell immunological parameters underlying this variability, we first examined, in the same pediatric cohort (Table S1), the relationship between neutralization breadth and the frequency of circulating T_FH_ cells in ART-naïve children (defined as CD3^+^CD4^+^CD45RA^−^CXCR5^+^CXCR3^−^PD1^+^ lymphocytes) (Figure 1A), cells which have previously been linked with HIV neutralization breadth in adults (44, 45). In these children also, we now observed a clear positive correlation between neutralization breadth and circulating “effector T_FH_” frequency (CCR7^−^; *r* = 0.44, *p* = 0.007), but no association with “central T_FH_” frequency (CCR7^+^; Figure 1B), consistent with previous studies showing that only circulating T_FH_ cells expressing an effector phenotype are functional active (46). Interestingly, there is a significant correlation between the frequency of central T_FH_ and viral load (*r* = 0.5, *p* = 0.003), but not with the key effector T_FH_ subset (Figure S1A). Additionally, PD1 expression on total CD4 and CD8 T-cells, which is linked to viral load and immune activation in HIV infection, is inversely correlated with neutralization breadth (Figure S1B). Taken together, these data suggest the observed association between the frequency of circulating effector T_FH_ and the development of broadly neutralizing antibodies is not driven by viremia or immune activation. Finally, consistent with the importance of the effector T_FH_ subset, circulating effector memory T_FH_ but not central memory T_FH_ were substantially more abundant in HIV-infected children compared to HIV-infected adults (effector memory T_FH_ median 4.9 vs. 2.7% of CD4 T-cells, *p* = 0.004; Figure 1C).

### T_FH_ are more abundant in lymphoid tissue of children than adults

**Figure 2:**
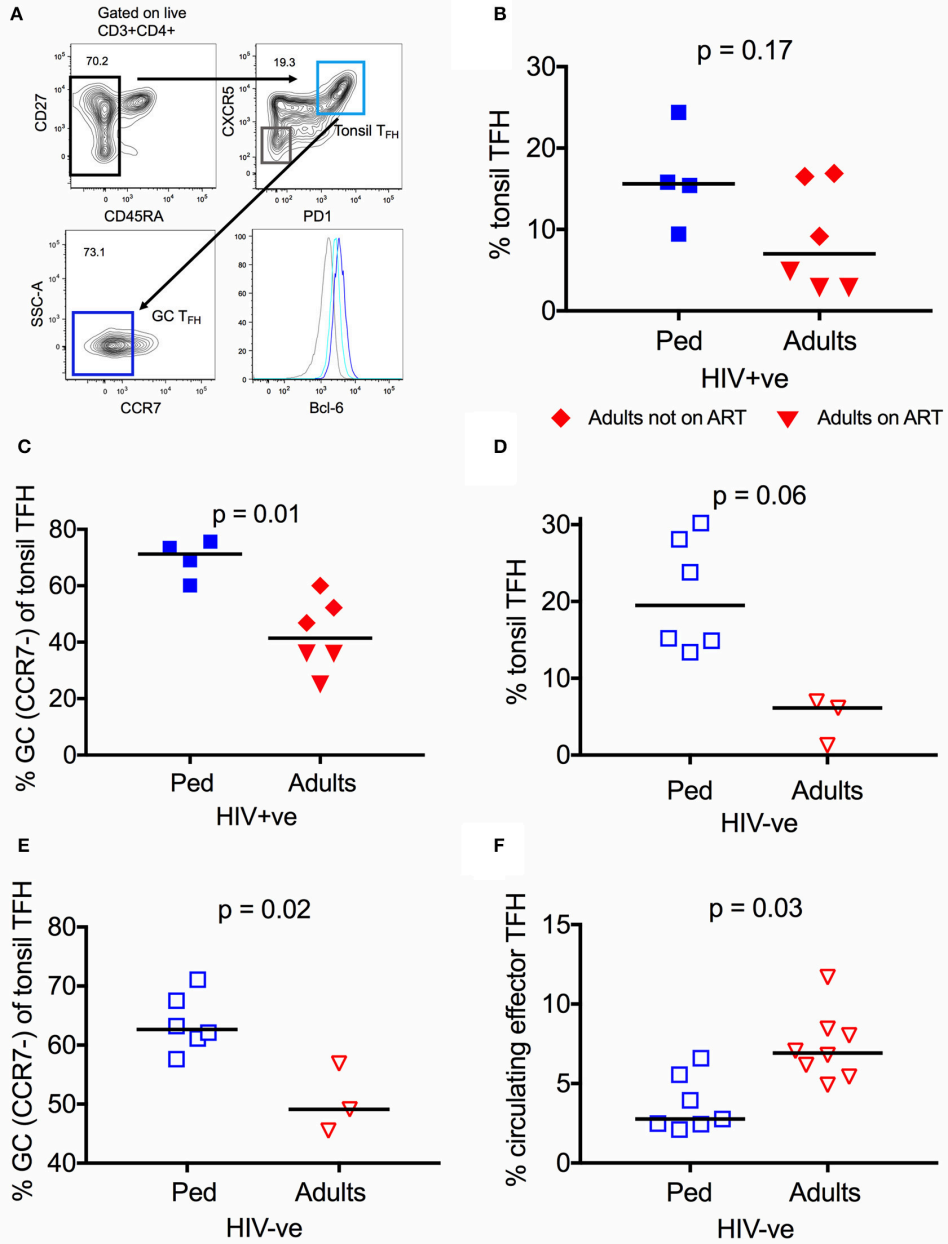
T_FH_ are more abundant in lymphoid tissue of children than adults. **(A)** Gating strategy of tonsil T_FH_ (CD4^+^CD45RA^−^CXCR5^+^PD1^high^; light blue) and GC-T_FH_ cells (%CCR7^−^ of total tonsil T_FH_; dark blue) in secondary lymphoid tissue. Levels of Bcl-6 expression of the different subsets are expressed in MFI (median fluorescent intensity). **(B)** No significant difference in the frequency of tonsil T_FH_ in secondary lymphoid tissue of infected children (blue squares; *n* = 4) compared to infected adults (n.s.; Kruskal-Wallis test). Red triangles: Adults on ART (*n* = 3), red diamonds: Adults not on ART (*n* = 3). **(C)** Same as in **(B)** but showing a significantly increased frequency of GC-T_FH_ cells (% CCR7^−^ of tonsil T_FH_) (*p* = 0.01; Kruskal-Wallis test) in infected children. **(D)** Increased frequency of tonsil T_FH_ (*p* = 0.06; Kruskal-Wallis test) in secondary lymphoid tissue of uninfected children (open blue squares; *n* = 6) compared to uninfected adults (open red triangles; *n* = 3). **(E)** Same as **(D)** but showing the increased frequency of GC-T_FH_ cells in uninfected children (*p* = 0.02; Kruskal-Wallis test) compared to uninfected adults. **(F)** Increased frequency of circulating effector T_FH_ in HIV uninfected adults (open red triangles; *n* = 8) compared to uninfected children (open blue squares; *n* = 7) (*p* = 0.03; Kruskal-Wallis test). In scatter plots medians are shown.

In order to investigate T_FH_ cells within lymphoid tissue, where their function is primarily exerted, we next studied tonsils isolated from HIV infected adults (*n* = 6) and children (*n* = 4, age median 11.1, see methods for cohort description). The six adults studied comprised 3 receiving ART and 3 not receiving ART (median viral load 2452 HIV RNA cp/ml), whilst all 4 children were receiving ART (median viral load 54 HIV RNA cp/ml) (Table S1) due to lack of sample availability of an ART-naïve pediatric control group. Plasma neutralization data of these individuals is not available, since the neutralization assays cannot be performed with detectable drug plasma levels. Hence, no direct associations to neutralization breadth can be drawn. In contrast to T_FH_ in circulation, lymphoid T_FH_ express the canonical transcriptional factor Bcl-6, which was used to confirm the identity of the subsets studied (Figure 2A). We observed that tonsil T_FH_ were increased in frequency in HIV infected children compared to adults, although this does not reach statistical significance (15.6 vs. 7.0%, *p* = 0.17; Figure 2B). T_FH_ cells up-regulate CXCR5 and PD1, and down-regulate CCR7 (47, 48) during migration from the T cell zone into the GC. When looking at “germinal center” T_FH_ (GC T_FH_; % CCR7^−^ of tonsil T_FH_) we found that they are enriched in HIV infected children compared to adults (Median 71.2 vs. 41.4%, *p* = 0.01; Figure 2C). Thus, HIV infected children have significantly higher levels of both circulating effector T_FH_ in the blood and GC T_FH_ from oral mucosal lymphoid tissue compared to infected adults, supporting the hypothesis that these cells contribute to HIV bnAb development.

To investigate whether these differences are observed also in HIV uninfected individuals, we next examined tonsillar lymphoid tissue in uninfected adults (*n* = 3) and children (*n* = 6). As in the HIV infected counterparts, the frequency of T_FH_ is higher in uninfected children compared to adults (Figures 2D,E), particularly for GC T_FH_ (62.7 vs. 49.1%, *p* = 0.02; Figure 2E). Unexpectedly, however, circulating effector T_FH_ cells were found to be significantly less frequent in uninfected children (*n* = 7) vs. adults (*n* = 8; *p* = 0.03; Figure 2F). Taken together, these data indicate that children possess more abundant GC T_FH_ to support the generation of neutralization breadth.

### High-frequency IL-21 production by HIV-specific GC-T_FH_ cells in children but not adults

**Figure 3:**
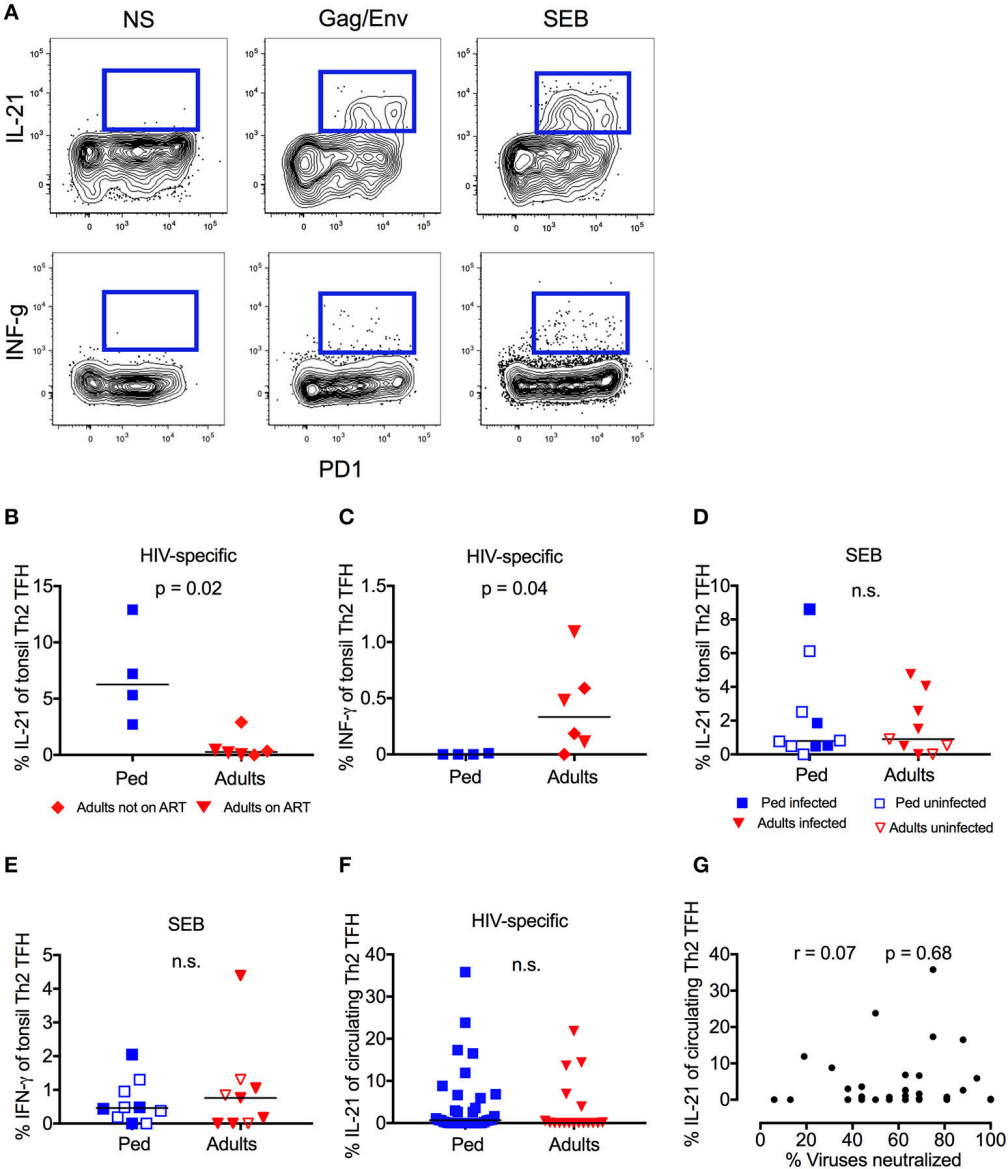
High-frequency IL-21 production in HIV-specific GC-T_FH_ Cells in children but not adults. **(A)** Representative flow cytometry plot of one pediatric HIV-infected subject. Gated on CD4^+^CCR6^−^CXCR3^−^CXCR5^+^ “Th2” T_FH_ cells. IL-21 (top row) and INF-γ (bottom row) secretion of PD1+ve cells is shown in response to HIV-peptide pools (middle) or SEB (right). On the left unstimulated control. **(B)** Gated on tonsil “Th2” GC-T_FH_ cells as follows: CD4^+^CCR6^−^CXCR3^−^CXCR5^+^PD1^+^. Increased IL-21 secretion (*p* = 0.02) and **(C)** decreased INF-γ secretion (p = 0.04) in HIV infected children (blue squares; *n* = 4) compared to infected adults in response to Gag/Env. Red triangles: Adults on ART (*n* = 3), red diamonds: Adults not on ART (*n* = 3). Mann-Whitneys test was used for comparisons between 2 groups. **(D)** No significant differences of IL-21 or INF-γ **(E)** production in response to SEB in children (blue; *n* = 10) compared to adults (red; *n* = 9) irrespective of HIV infection and ART status (n.s.; Mann-Whitneys-test). Closed symbols: HIV infected; open symbols: HIV uninfected. **(F)** No significant differences in Gag/Env specific IL-21 secretion of circulating “Th2”-T_FH_ cells between ART-naïve infected children (blue squares; n = 38) and ART-naïve infected adults (red triangles; *n* = 18) (n.s.; Kruskal-Wallis test). **(G)** Lack of correlation between HIV-specific (Gag/Env pool) IL-21 production of circulating “Th2”-T_FH_ and neutralization breadth in HIV-infected, ART-naïve children (*n* = 36). Calculations were made by Spearman's rank correlation test. In scatter plots medians are shown.

Germinal center (GC) T_FH_ cells promote B-cell proliferation, somatic hypermutation, and affinity maturation through IL-21 production, and this function is therefore critical for the generation of bnAbs (49–51). In response to stimulation with peptide pools spanning Gag and Env, “Th2”-GC-T_FH_ cells (by far the dominant T_FH_ subset present in tonsil homogenate Figure S2A) from HIV infected children made strong IL-21 responses (median 6.3%), whereas “Th2”-GC-T_FH_ cells from HIV-infected adults produced little or no IL-21 (Figures 3A,B). By contrast, the reverse was the case with respect to IFN-γ production, which was detectable in adults (median 0.3%) but absent in children (Figure 3C). These differences between children and adults were both statistically significant (*p* = 0.02 and *p* = 0.04, respectively) despite the small sample size, and the same trend was observed for total cytokine producing CD4 cells (Figures S2B,C). In response to stimulation by SEB, however, there was no difference in the responses made by “Th2”-GC-T_FH_ cells from children or adults, irrespective of HIV infection (Figures 3D,E), indicating that the differences observed between children and adults were among HIV-specific GC-T_FH_ cells. Importantly, the bias in IL-21 and INF-γ production between adults and children observed in HIV-specific GC-T_FH_ was not seen in circulating “Th2”-T_FH_ cells (Figure 3F, Figure S2D). Consistent with this, we found no correlation between HIV-specific IL-21 production by circulating “Th2”-T_FH_ in ART-naïve children and neutralization breadth (Figure 3G). Thus, specifically in the germinal centers of lymphoid tissue, both a greater abundance of T_FH_ and a higher frequency of HIV-specific T_FH_ that secrete the key functional cytokine IL-21 were observed in HIV-infected children compared to adults.

### Tonsil follicular regulatory T cells (T_FR_) are increased in HIV-infected children

**Figure 4:**
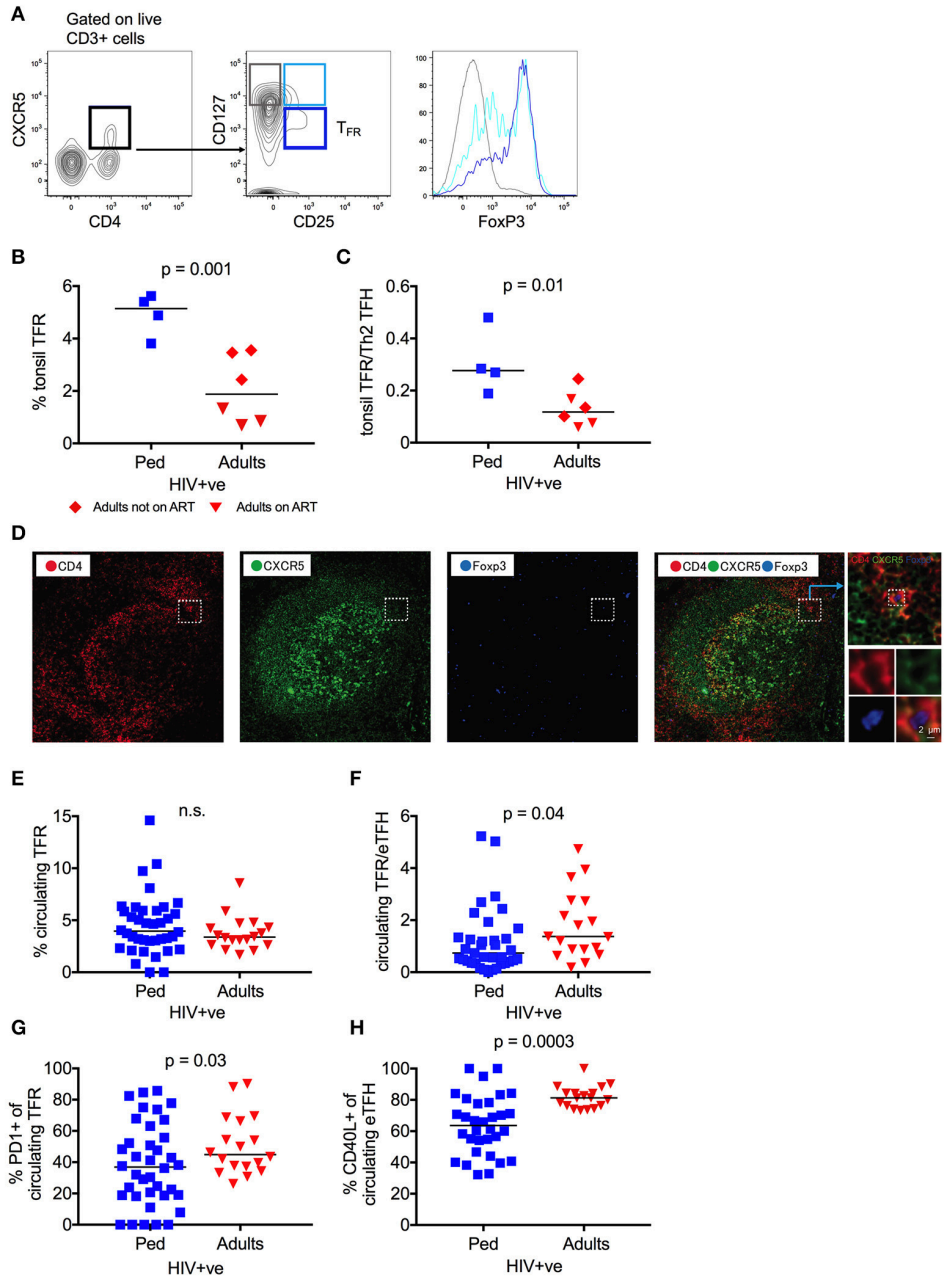
Tonsil follicular regulatory T cells (T_FR_) are increased in HIV infected children. **(A)** CD4^+^CXCR5^+^CD25^+^CD127^low^ follicular regulatory T cells (T_FR_) (dark blue) show the highest FoxP3 expression as expressed in MFI (median fluorescent intensity) when compared to CD4^+^CXCR5^+^CD25^+^CD127^high^ (light blue) and CD4^+^CXCR5^+^CD25^−^CD127^high^ (gray) non-regulatory follicular T cells. **(B)** Increased frequency of tonsil T_FR_ in HIV infected children (blue squares; *n* = 4) compared to infected adults (red triangles: Adults on ART, n = 3; red diamonds: Adults not on ART, *n* = 3) (*p* = 0.001; Kruskal-Wallis test). **(C)** Same as B but showing the increased ratio of tonsil T_FR_ to tonsil “Th2” T_FH_ cells (gated on CD4^+^CCR6^−^CXCR3^−^CXCR5^+^PD1^+^) in HIV infected children compared to infected adults (*p* = 0.01; Kruskal-Wallis test). **(D)**. Immunofluorescent staining of a tonsil sample from which histological sections were available confirms the existence of CXCR5^+^ FoxP3^+^ CD4 T-cells in proximity to the GC. CD4: red; CXCR5: green; FoxP3: blue. **(E)** No differences in frequency of circulating T_FR_ between infected, ART-naïve children (blue squares; *n* = 38) and ART-naïve adults (red triangles; n = 18) (n.s.; Kruskal-Wallis test). **(F)** Same as F but showing an increased ratio of circulating T_FR_ to circulating effector T_FH_ (gated on CD4^+^CD45RA^−^CXCR5^+^CXCR3^−^CCR7^−^PD1^+^) in HIV infected adults compared to infected children (p = 0.04; Kruskal-Wallis test). **(G)** Increased expression of PD1 on circulating T_FR_ of HIV infected adults compared to HIV infected children (*p* = 0.03; Kruskal-Wallis test). **(H)** Decreased expression of CD40L on circulating effector T_FH_ cells in children when compared to adults (*p* = 0.0003; Kruskal-Wallis test). In scatterplots, medians are shown.

T_FH_ cells are needed to mediate an optimal antibody response, but excessive T_FH_ activity leads to the expansion of low-affinity and auto reactive B cells (24, 25, 52). T-follicular regulatory cells (T_FR_), a CD4 T cell subset that regulates germinal center T_FH_ responses, are thus essential for the generation of high affinity antibodies (26, 27, 33). Therefore, we next examined the relationship between T_FH_ and T_FR_ within HIV infected tonsils, defining T_FR_ as CD4^+^CXCR5^+^CD25^+^CD127^low^ cells that are enriched for the T_REG_ transcriptional factor FoxP3 (30) (Figure 4A), and having found that CD25 expression is not affect by HIV infection (Figure S3A). In HIV infected children, the T_FR_ frequency was 2.7-fold higher than that observed in HIV-infected adults (median 5.1 vs. 1.9%; *p* = 0.001), particularly those adults on ART (Figure 4B). This profound enrichment of T_FR_ results in a significantly higher T_FR_/T_FH_ ratio in HIV infected children, despite the increased frequency of T_FH_ in this group (*p* = 0.01; Figure 4C). Immunofluorescent staining of a tonsil sample from which histological sections were available confirms the existence of CXCR5^+^ FoxP3^+^ CD4 T-cells in proximity to the GC (Figure 4D). In contrast to T_FH_, however, this trend is not observed in HIV uninfected counterparts, in whom T_FR_ frequency and T_FR_/T_FH_ ratio are not significantly different (Figures S3B,C). Overall, these data suggest that the enriched and functionally superior GC T_FH_ in HIV-infected children are also better regulated than in HIV-infected adults, which is likely to support affinity maturation and the development of bnAbs.

Interestingly, no differences between HIV-infected children and adults were observed in circulating T_FR_ frequency, and the ratio of circulating T_FR_ to effector T_FH_ cells was significantly *lower* in HIV-infected, ART-naïve children compared to adults (*p* = 0.04; Figures 4E,F and Figures S3D,E). However, circulating T_FR_ from HIV-infected adults express significantly higher levels of the exhaustion marker PD1, known to impair T_FR_ function (*p* = 0.03; Figure 4G) (53). Furthermore, circulating pediatric T_FH_ expressed lower levels of the surface marker CD40L (*p* = 0.0003; Figure 4H), which implies tighter regulation through this key T_FH_ functional molecule. Thus, although the frequency of circulating T_FR_ is lower in HIV-infected children, in contrast to the frequency of GC T_FR_, the phenotypic differences observed between children and adults are consistent with increased regulation of the T_FH_ response in HIV infected children in circulation as well as in lymphoid tissue. This is corroborated by a positive trend between the frequency of circulating T_FR_ and neutralization breadth in HIV infected children and negative trend in the same group between PD1 expression and breadth (Figures S3F,G). Again, these data support the importance of T_FH_ regulation in the preferential development of bnAbs in HIV-infected children.

### Follicular CD8 T-cells correlate with neutralization breadth in children

**Figure 5:**
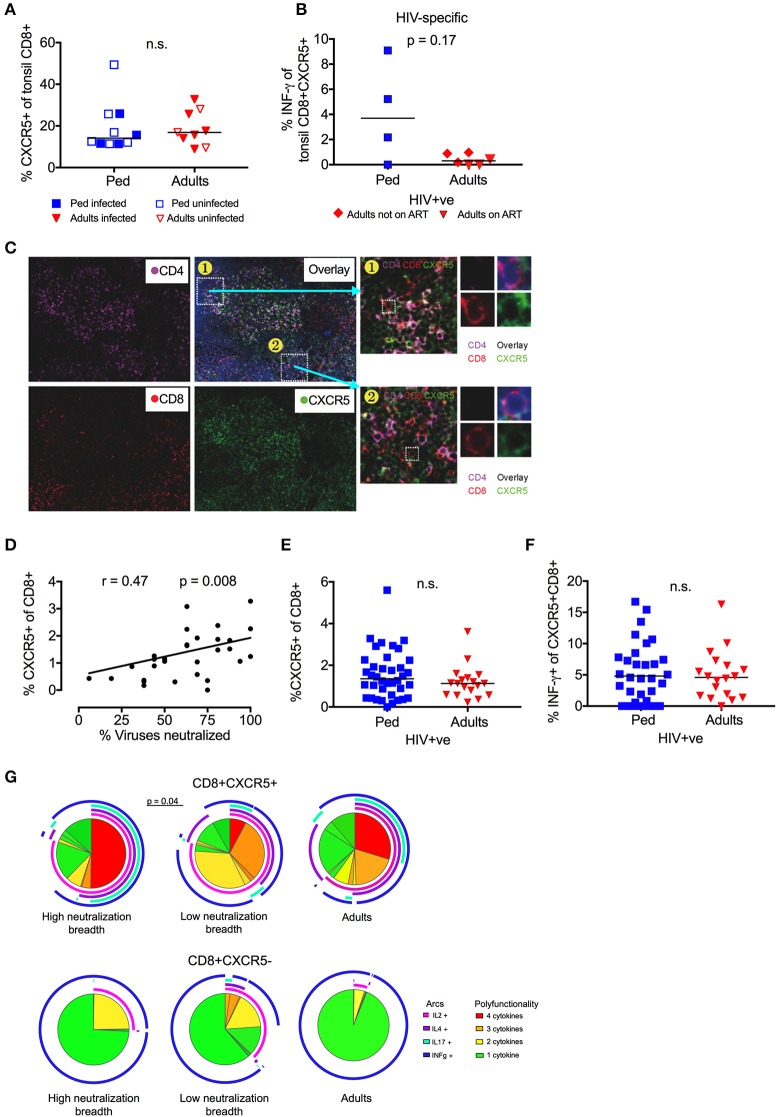
Follicular CD8 T-cells correlate with neutralization breadth in HIV infected children. **(A)** No significant difference in frequency of tonsil CXCR5+ve CD8 T-cells between children (blue squares; n = 10) and adults (red triangles; *n* = 9) irrespective of HIV infection (closed symbols: HIV infected; open symbols: HIV uninfected) (n.s; Mann-Whitney test). **(B)** Increased secretion of INF-γ of tonsil CXCR5+ve CD8 T-cells in response to Gag/Env pool in HIV-infected pediatric study participants (*n* = 4) compared to infected adults (red triangles: Adults on ART, *n* = 3; red diamonds: Adults not on ART, *n* = 3) (*p* = 0.17; Mann-Whitneys test). **(C)** Immunofluorescent staining of available histological sections shows the presence of CXCR5+ve CD8 T-cells in association with the GC. CD4: violet; CD8: red; CXCR5: green. **(D)** Frequency of circulating CXCR5+ve CD8 T cells correlates with neutralization breadth within the cohort of HIV infected children (*p* = 0.008, *r* = 0.47; Spearman's rank test) (*n* = 36). **(E)** No significant differences in the frequency of circulating CXCR5+ve CD8 T-cells or HIV-specific (Gag/Env pool) INF-γ production **(F)** between HIV-infected, ART-naïve children (blue squares; *n* = 38) and adults (red triangles; *n* = 18). Comparisons between 2 groups were performed using Mann-Whitneys test and between >2 groups using Kruskal-Wallis-test and corrected for multiple comparisons. **(G)** Circulating CXCR5+ve CD8 T-cells (left) show a more polyfunctional cytokine response to HIV Gag/Env compared to CXCR5–ve CD8 T-cells (right). Circulating CXCR5+ve CD8 T-cells of children with high neutralization breadth show a stronger polyfunctional profile than those of children with low neutralization breadth (*p* = 0.04) (Monte Carlo simulation partial permutation test). Cytokine-negative cells are excluded from the pie chart. (color coding pie chart: red: 4 cytokines, orange: 3 cytokines, yellow: 2 cytokines, green: 1 cytokine; color coding arcs: pink: IL-2, violet: IL-4, turquoise: IL-17, blue: INF-γ).

To further explore the contribution of T_FH_ regulation to bnAb development, we next studied CXCR5+ve CD8 T-cells, a subset also known to limit GC T_FH_ cell activity (35, 36, 38, 40, 54). The overall frequency of CXCR5+ve CD8 T-cells in tonsils did not differ between children and adults, in either HIV infected or uninfected individuals (Figure 5A). However, HIV-specific CXCR5+ve CD8 T-cells that produced IFN-γ in response to stimulation with Gag/ Env peptide pools were of considerably higher magnitude (median 3.7 vs. 0.3%) in infected children compared to adults, although this did not reach statistical significance (*p* = 0.17; Figure 5B). Immunofluorescent staining of available histological sections confirmed the presence of CXCR5+ve CD8 T-cells in association with CD4 T-cells within the GC (Figure 5C). In circulation, we observed a significant positive correlation between the frequency of CXCR5+ve CD8 T-cells and neutralization breadth in HIV infected, ART-naïve children (*r* = 0.47, *p* = 0.008; Figure 5D). As with circulating T_FR_, the frequency of total CXCR5+ve CD8 T-cells was similar in infected adults and children, as was the frequency of HIV-specific CXCR5+ve CD8 T-cells (Figures 5E,F). However, CXCR5+ve CD8 T-cells were significantly more polyfunctional in children with high neutralization breadth compared to those with low neutralization breadth (*p* = 0.04; Figure 5G) (functions tested being IL-2, IL-4, IL-17 and IFN-γ production in response to HIV Gag/ Env). These differences in functionality were not observed in CXCR5-ve CD8+ T-cells and no significant differences were seen between adults and children as a whole. Together, these data support a role for CXCR5+ve CD8 T-cells, in addition to T_FR_ in the regulation of T_FH_ in HIV-infected children.

## Discussion

Understanding the immunological conditions in which bnAbs against HIV-1 are generated will help to optimize and target future vaccine strategies. Although most HIV-infected children produce broad and potent neutralizing antibodies against HIV-1 (17, 18), the relationship between the T_FH_ response, which is critical for affinity maturation, and neutralization breadth has not been investigated to date in children. The need to examine lymphoid tissue in particular was made apparent by the current study, as many of the key differences in T_FH_ activity between children and adults were only evident within the tonsil. Circulating effector T_FH_ were more frequent in infected, ART-naïve children than adults and correlate well with neutralization breadth; and GC T_FH_ in tonsil were more than double the frequency in children. However, differences between children and adults in antigen specific T_FH_ were only observed in lymphoid tissue. HIV-specific “Th2”-GC T_FH_ were more abundant in children and produced IL-21 and not IFN-y, whereas adult HIV-GC T_FH_ cells secreted IFN-y not IL-21. Additionally, these studies of lymphoid tissue underline the key role of T_FH_ regulation in the development of HIV bnAbs in children, as T_FR_ cells were present at substantially higher frequencies than in infected adults, giving rise to significantly higher T_FR_/T_FH_ ratios in HIV-infected but not in uninfected children. Furthermore, HIV-specific CXCR5+ CD8 T-cells, described to have regulatory function in the GC (36, 39, 54), were more frequent in pediatric lymphoid tissue; and total circulating CXCR5+ve “follicular” CD8 T-cells correlated with neutralization breadth and were more polyfunctional in children with high neutralization breadth. Together these data are consistent with the notion that increased HIV-specific T_FH_ activity and increased regulation within GC both contribute to the high frequency of potent, bnAbs observed in HIV-infected children.

In response to stimulation by HIV peptides, peripheral blood T_FH_ cells produced very low levels of the canonical T_FH_ cytokine, IL-21 (55), and no correlation with antibody breadth or differences between children and adults were observed. These children were not on ART at the time of sampling, which reduces circulating HIV-specific T-cells (56). Thus, in children at least, limited information on HIV-specific T_FH_ can be obtained from the blood. This contrasted with the striking HIV-specific IL-21 Th2 T_FH_ responses in HIV infected pediatric tonsils and their almost complete absence in the tonsils of the adults tested. T_FH_ provide help to B-cells in an antigen-specific manner (57), and hence the presence of HIV-specific T_FH_ secreting the appropriate T_FH_ cytokines is likely to be critical to effective affinity maturation of HIV antibodies. The GC HIV-specific T_FH_ responses in adults were both less abundant and functionally distinct in secreting IFN-γ rather than IL-21. The Th2 T_FH_ bias observed here in HIV-infected children, compared with adults, and the more frequent development of bnAbs in children is consistent with other recent findings. In HIV-infected adults, IFN-γ secreting T_FH_ did not support antibody class switching as effectively as Th2 biased T_FH_ secreting Th2 cytokines (58). Furthermore, in the SIV model, Th2-skewed T_FH_ correlated with the development of broadly neutralizing antibodies, whereas IFN-γ producing Th1 T_FH_ did not (59). Other studies in both humans and non-human primates point to the overall Th1 polarization of the GC T_FH_ response in chronic infection that impairs optimal B-cell activity (60, 61). Together, these data support the hypothesis that the HIV-specific IL-21-producing T_FH_ within the lymphoid tissue of HIV-infected children play a critical role in the generation of the high-frequency, potent bnAbs that are characteristically observed in children. The mechanism by which these differences arise remains unclear, especially since responses to SEB did not differ between adults and children. It is possible that the increased frequency of T_FH_ observed in children reflects recent exposure to other common childhood infections. Indeed, this is consistent with the increased frequency of GC T_FH_ observed in the tonsils of HIV uninfected children compared to adults. However, both the correlation between T_FH_ frequency and neutralization breadth, and the existence of high frequency HIV-specific GC T_FH_, demonstrates a significant HIV-specific component to this trend.

The other key difference between HIV infected children and adults highlighted by this study is the evidence of greater regulation of the T_FH_ response in children. Again, this is most apparent in the lymphoid tissue, where regulation of T_FH_ activity is most relevant (25). This makes biological sense, as the absence of T_FR_ has been shown to cause an outgrowth of non-antigen specific or low-affinity B cells in germinal centers and leads to fewer antigen-specific cells (23, 27). Other work has suggested that expansion of regulatory cells in the GC in adult HIV and SIV infection may inhibit the generation of bnAbs (62). In addition, several studies have linked HIV bnAb development to a loss of immune tolerance and to autoimmunity (63–66), and reduced circulating T_REG_ (66). This has led to the hypothesis that bnAb development is constrained by host tolerance controls and that lower regulation would promote the development of breadth. However, this concept has been based exclusively on work in adult infection, and this present study highlights the immune differences between HIV infected adults and children (4). For example, although we observed a similar increase in circulating T_FH_ in children and adults, pediatric T_FH_ expressed significantly lower levels of CD40L, which is crucial for providing B-cell help. Neonatal T cells are known to express lower levels of CD40L than adult counterparts (67), and these levels appear to increase with age (68). Overexpression of CD40L can lead to hypergammaglobulinemia in a lymphopenic mouse model (52), a characteristic of untreated HIV infection in adults that is associated with T_FH_ activity (48). Therefore, lower expression of CD40L on pediatric T_FH_ may help to regulate these cells as the developing immune system is exposed to new antigens and facilitate the development of bnAbs. Whether children make superior antibodies to adults *per se* is unclear and is complicated both by exposure that, for most common infections, occurs in childhood, and the influence of maternal antibodies. Infants infected with Rota virus, display superior neutralizing antibody responses greater than their mothers (69), and potent cross-clade neutralizing antibody responses against Enterovirus exist in children (70). However, children do not appear to make neutralizing antibodies to Influenza A H5/N1 infection, whilst adults do (71). Further study is required to better understand the process of antibody development in children, which may vary depending on pathogen, route and timing of infection.

It is important to highlight that tonsil samples studied here are not from the same subjects studied previously and from which the neutralization data derive (17). Moreover, it was not possible to obtain neutralization data from these children, due to ongoing ART (72). However, given that the ability of typical, progressing HIV infected children to neutralize a broad range of viruses is so much greater than that of HIV infected adults [a median of 63% of viruses neutralized vs. 25% of the same test set of viruses, respectively, *p* < 0.0001, (17)], it is clear that children are fundamentally different from adults in this respect. There is no reason to suppose that the children and adults studied here are not representative of those in whom neutralizing antibody responses have been previously mapped. The impact of ART on tonsil T_FH_ and regulatory T-cell frequency and function within infected children is unclear. Limited studies indicate that ART treatment of chronically infected adults can reduce the frequency of T_FH_ within lymph nodes, though it remains higher than uninfected adults (73). This is consistent with our own data in which the 3 ART treated adults have fewer tonsil T_FH_ and T_FR_ than the 3 untreated individuals. Indeed, if one considers only ART treated individuals, the difference in frequency of both cell types is even greater between children and adults.

Taken together, these data suggest, in children, the immune system is well adapted to the production of broadly HIV neutralizing antibodies, likely as a result of the immunotolerant immune environment in early life (74–76), that includes a relatively high degree of regulation of a high-frequency T_FH_ response. In adults, where T_FH_ cells appear to be substantially less abundant, better generation of bnAbs may depend on the strategy of decreasing T_FH_ regulation (66). However, given the inherent aggressive nature of the adult immune response, decreasing T-cell regulation to increase bnAb generation may simultaneously bring the unwanted consequences of increased autoimmunity. Children, by contrast, may represent a much more attractive group in which long lasting broadly neutralizing antibodies against HIV infection are more easily generated, thereby providing protection against HIV infection when they become adults.

## Author contributions

JR designed the study, conducted experimental work within the study, analyzed the data, and wrote the paper. TM conducted experimental work within the study and analyzed the data. AN, DR, MM, EA, TA, and SK conducted experimental work within the study. WK, TN, FK, and PJ designed the study, recruited the subjects, and analyzed the data. AS, LM, PM, and SP conducted/supervised experimental work within the study. HK, PG, and AL supervised experimental work within the study, analyzed data, and wrote the paper. PG and AL established research cohorts.

### Conflict of interest statement

The authors declare that the research was conducted in the absence of any commercial or financial relationships that could be construed as a potential conflict of interest.

## Supplementary material

Figure S1 No correlation between viral load and effector T_FH_ frequency. **(A)** Frequency of circulating central T_FH_ correlates significantly with viral load within the pediatric cohort (*r* = 0.5, *p* = 0.0003; left) while there is no correlation with circulating effector T_FH_ frequency (right). **(B)** Inverse correlation between PD1 expression on bulk CD4 (left) and CD8 (right) T-cells and viral load within the pediatric cohort. All correlations are performed by Spearman's rank test (*n* = 36).

Figure S2 High-frequency IL-21 production in HIV-specific GC-T_FH_ cells in children but not adults. **(A)** The majority of tonsil T_FH_ cells are “Th2”-T_FH_ cells (CD4^+^CCR6^−^CXCR3^−^CXCR5^+^PD1^+^). See gating strategy on the left and distribution of Th2 (CCR6^−^CXCR3^−^), Th17 (CCR6^+^CXCR3^−^) and Th1 (CCR6^−^CXCR3^+^) of tonsil T_FH_ on the right. **(B)** Tonsil bulk CD4 T-cells of infected children (blue squares; *n* = 4) secrete more IL-21 and less INF-g **(C)** in response to HIV peptide than CD4 T cells of infected adults (red triangles: Adults on ART, *n* = 3; red diamonds: Adults not on ART, *n* = 3) (n.s., Mann–Whitneys test). **(D)** No significant differences in Gag/Env specific IL-21 secretion of circulating “Th2”-T_FH_ cells between ART-naïve infected children (blue squares; *n* = 38) and ART-naïve infected adults (red triangles; *n* = 18) (n.s., Kruskal–Wallis test). In scatterplots, medians are shown.

Figure S3 Tonsil follicular regulatory T cells (T_FR_) are increased in HIV-infected children. **(A)** Similar expression of CD25 in HIV infected and uninfected children (left: exemplary FACS plot; right: summary data of all pediatric samples). Closed blue squares: HIV infected (*n* = 38), open blue squares: HIV uninfected (*n* = 7). **(B)** No significant differences in the frequency of tonsil T_FR_ or ratio of tonsil T_FR_ to tonsil “Th2” T_FH_ cells (gated on CD4^+^CCR6^−^CXCR3^−^CXCR5^+^PD1^+^) **(C)** in uninfected children (open blue squares; *n* = 6) compared to uninfected adults (open red triangles; *n* = 3) (n.s., Kruskal–Wallis test). **(D)** No significant differences in the frequency of circulating T_FR_ in uninfected children (open blue squares; *n* = 7) compared to adults (open red triangles; *n* = 8) (n.s., Kruskal–Wallis test). **(E)** Uninfected children shown an increased ratio of circulating T_FR_ to circulating effector T_FH_ (gated on CD4^+^CD45RA^−^CXCR5^+^CXCR3^−^CCR7^−^PD1^+^) compared to uninfected adults (*p* = 0.02; Kruskal–Wallis test). **(F)** Positive non-significant correlation of frequency of circulating T_FR_ and neutralization breadth in HIV-infected children (*r* = 0.29, *p* = 0.09; Spearman's rank test) (*n* = 36). **(G)** Inverse non-significant correlation of PD1 expression on circulating T_FR_ and neutralization breadth within the pediatric cohort (*r* = −0.3, *p* = 0.08; Spearman's rank test) (n = 36). Medians are shown in scatter plots.

Table S1 Clinical characteristics of study cohort.

Table S2 List of antibodies for flow cytometry.
