# Supplementary figures and images for: High-Frequency, Functional HIV-Specific T-Follicular Helper and Regulatory Cells Are Present Within Germinal Centers in Children but Not Adults
Source: Front Immunol. 2018 Sep 12;9:1975. doi: 10.3389/fimmu.2018.01975 (PMC6143653; doi:10.3389/fimmu.2018.01975)

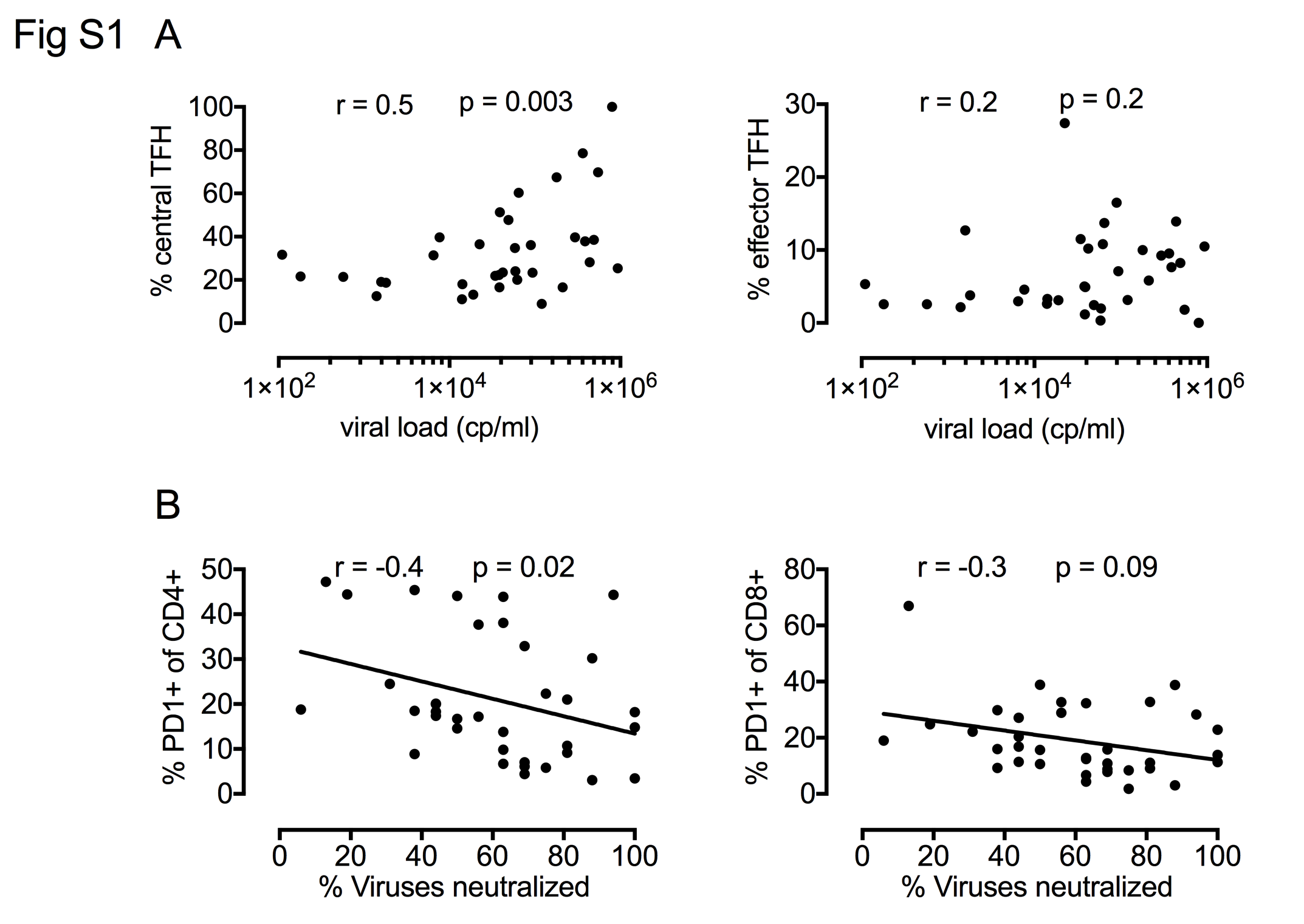

Supplement: Figure S1 — No correlation between viral load and effector TFH frequency. (A) Frequency of circulating central TFH correlates significantly with viral load within the pediatric cohort (r = 0.5, p = 0.0003; left) while there is no correlation with circulating effector TFH frequency (right). (B) Inverse correlation between PD1 expression on bulk CD4 (left) and CD8 (right) T-cells and viral load within the pediatric cohort. All correlations are performed by Spearman's rank test (n = 36). [file Image_1.tiff]

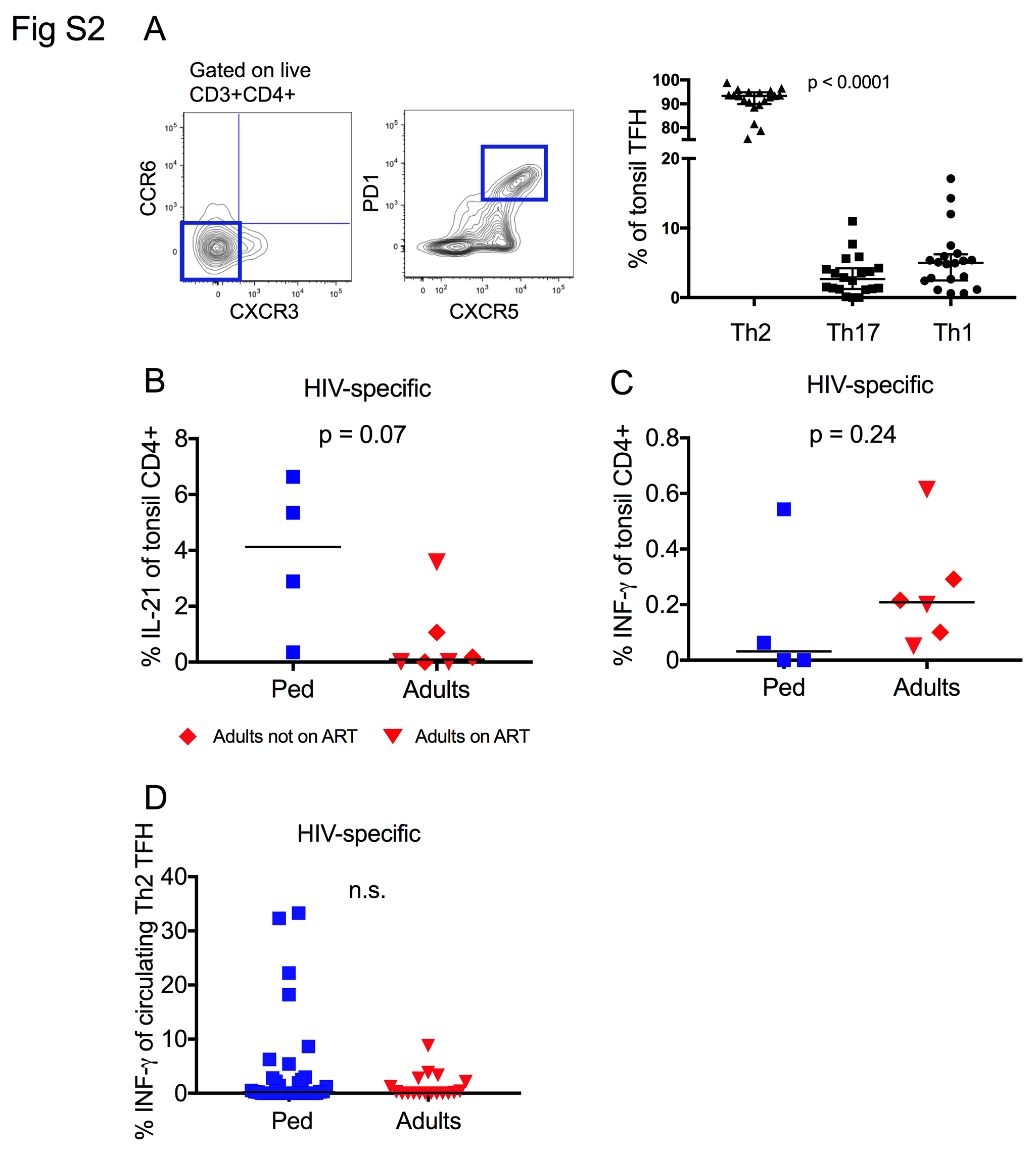

Supplement: Figure S2 — High-frequency IL-21 production in HIV-specific GC-TFH cells in children but not adults. (A) The majority of tonsil TFH cells are “Th2”-TFH cells (CD4+CCR6−CXCR3−CXCR5+PD1+). See gating strategy on the left and distribution of Th2 (CCR6−CXCR3−), Th17 (CCR6+CXCR3−) and Th1 (CCR6−CXCR3+) of tonsil TFH on the right. (B) Tonsil bulk CD4 T-cells of infected children (blue squares; n = 4) secrete more IL-21 and less INF-g (C) in response to HIV peptide than CD4 T cells of infected adults (red triangles: Adults on ART, n = 3; red diamonds: Adults not on ART, n = 3) (n.s., Mann–Whitneys test). (D) No significant differences in Gag/Env specific IL-21 secretion of circulating “Th2”-TFH cells between ART-naïve infected children (blue squares; n = 38) and ART-naïve infected adults (red triangles; n = 18) (n.s., Kruskal–Wallis test). In scatterplots, medians are shown. [file Image_2.tiff]

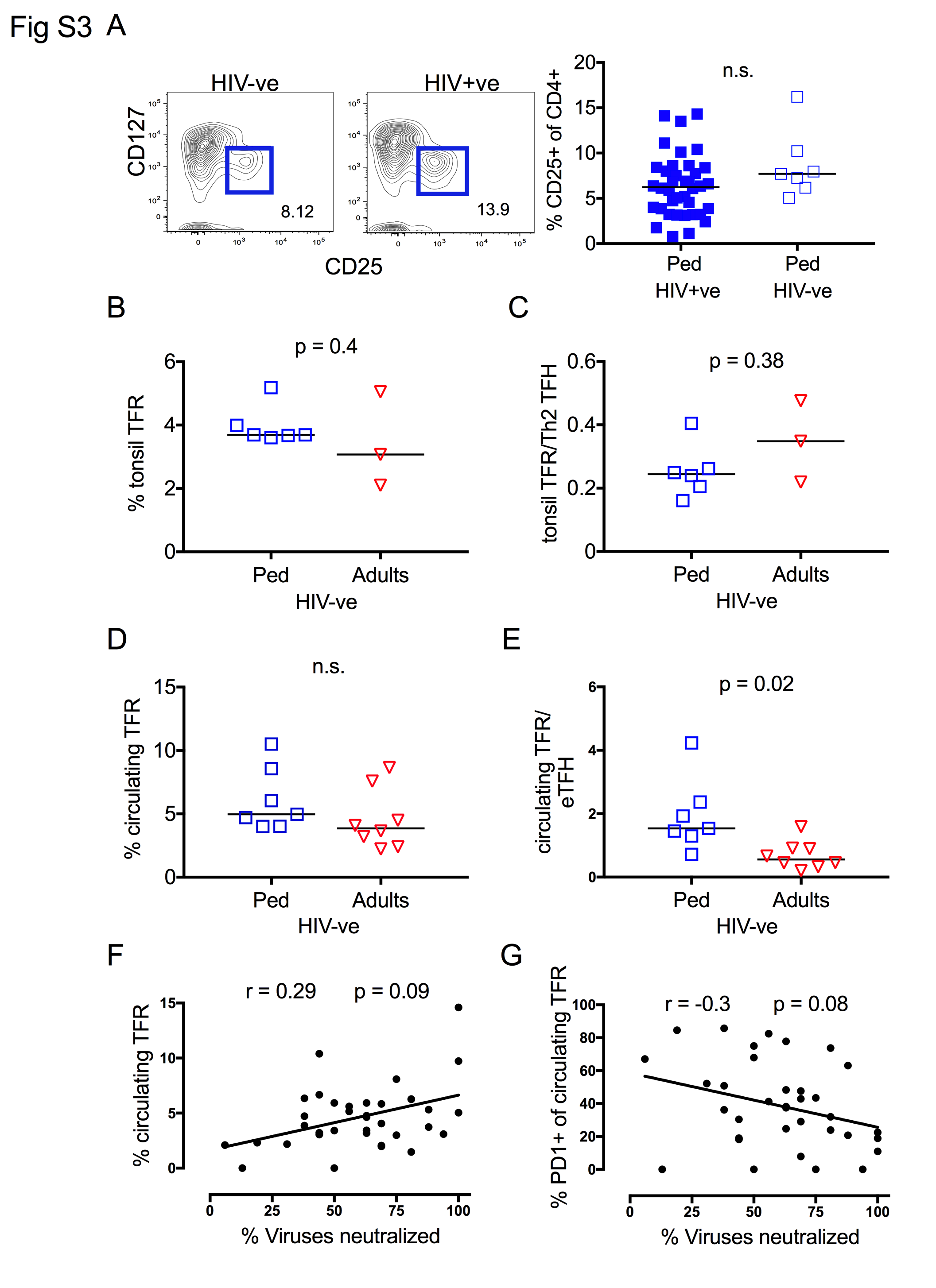

Supplement: Figure S3 — Tonsil follicular regulatory T cells (TFR) are increased in HIV-infected children. (A) Similar expression of CD25 in HIV infected and uninfected children (left: exemplary FACS plot; right: summary data of all pediatric samples). Closed blue squares: HIV infected (n = 38), open blue squares: HIV uninfected (n = 7). (B) No significant differences in the frequency of tonsil TFR or ratio of tonsil TFR to tonsil “Th2” TFH cells (gated on CD4+CCR6−CXCR3−CXCR5+PD1+) (C) in uninfected children (open blue squares; n = 6) compared to uninfected adults (open red triangles; n = 3) (n.s., Kruskal–Wallis test). (D) No significant differences in the frequency of circulating TFR in uninfected children (open blue squares; n = 7) compared to adults (open red triangles; n = 8) (n.s., Kruskal–Wallis test). (E) Uninfected children shown an increased ratio of circulating TFR to circulating effector TFH (gated on CD4+CD45RA−CXCR5+CXCR3−CCR7−PD1+) compared to uninfected adults (p = 0.02; Kruskal–Wallis test). (F) Positive non-significant correlation of frequency of circulating TFR and neutralization breadth in HIV-infected children (r = 0.29, p = 0.09; Spearman's rank test) (n = 36). (G) Inverse non-significant correlation of PD1 expression on circulating TFR and neutralization breadth within the pediatric cohort (r = −0.3, p = 0.08; Spearman's rank test) (n = 36). Medians are shown in scatter plots. [file Image_3.tiff]
