# Supplementary material for: High-Frequency, Functional HIV-Specific T-Follicular Helper and Regulatory Cells Are Present Within Germinal Centers in Children but Not Adults
Source: Front Immunol. 2018 Sep 12;9:1975. doi: 10.3389/fimmu.2018.01975 (PMC6143653; doi:10.3389/fimmu.2018.01975)
Supplement: Table S1 — Clinical characteristics of study cohort. [file Table_1.pdf]

Table S1

group	ART	compartment	n	age median (IQR)	$CD4/mm^3$ median (IQR)	VL cp/ml median (IQR)
Pediatric infected	no	PBMC	38	8.1 (6.6-9.8)	762 (297-1039)	55,000 (14,000-178,848)
Pediatric infected	yes	TMC	4	11.1 (9.4-12.1)	946 (532-1090)	54 (<50 - 5102)
Pediatric uninfected	N/A	PBMC	7	13.8 (12.0-15.0)	N/A	N/A
Pediatric uninfected	N/A	TMC	6	10.0 (7.8-11.5)	N/A	N/A
Adult infected	no	PBMC	18	29.5 (26.8-33.3)	356 (174-455)	56,000 (35,250-140,000)
Adult infected	no	TMC	3	27.0 (26.0-34.0)	493 (491-841)	2452 (1372-42,148)
Adult infected	yes	TMC	3	27.0 (26.0-31.0)	548 (379-591)	<50; N/D
Adult uninfected	N/A	PBMC	8	18.0 (17.0-19.8)	N/A	N/A
Adult uninfected	N/A	TMC	3	30.0 (28.3-34.8)	N/A	N/A

TMC: tonsil mononuclear cells
PBMC: peripheral blood mononuclear cells

N/A: not applicable
N/D: not done
