# Supplementary material for: High-Frequency, Functional HIV-Specific T-Follicular Helper and Regulatory Cells Are Present Within Germinal Centers in Children but Not Adults
Source: Front Immunol. 2018 Sep 12;9:1975. doi: 10.3389/fimmu.2018.01975 (PMC6143653; doi:10.3389/fimmu.2018.01975)
Supplement: Table S2 — List of antibodies for flow cytometry. [file Table_2.pdf]

Table S2

| antigen | fluorochrome | clone | supplier | panel |
|---|---|---|---|---|
| CD69 | BUV 395 | FN50 | BD | all TfH |
| CCR6 | BUV 496 | 11A9 | BD | all TfH |
| PD1 | BV 421 | EH12.1 | BD | all TfH |
| CD8 | V500 | RPA-T8 | BD | all TfH |
| CCR4 | BV 605 | L291H4 | biolegend | all TfH |
| CD45RA | BV 650 | HI100 | BD | pheno TfH |
| CD25 | BV 711 | BC96 | biolegend | pheno TfH |
| CD3 | BV 785 | OKT3 | biolegend | all TfH |
| CXCR5 | AlexaFluor 488 | RF8B2 | BD | all TfH |
| CCR7 | PerCp-Cy5-5 | G043H7 | biolegend | pheno TfH |
| CD127 | PE-Cy5 | R34.34 | Beckmann Coulter | all TfH |
| CD27 | PE-Cy7 | M-T271 | biolegend | pheno TfH |
| CXCR3 | PE-CF594 | 1C6/CXCR3 | BD | all TfH |
| CD40L | PE | 24-31 | biolegend | pheno TfH |
| ICOS | Alexa Fluor 647 | C398.4A | biolegend | all TfH |
| CD4 | AlexaFluor 700 | RPA-T4 | BD | all TfH |
| IL-2 | BV 650 | 5344.111 | biolegend | ICS TfH |
| INF-γ | BV 711 | 4S.B3 | biolegend | ICS TfH |
| IL-17 | PerCp-Cy5-5 | eBio64DEC17 | ebioscience | ICS TfH |
| IL-21 | PE | eBio3A3-N2 | ebioscience | ICS TfH |
| IL-4 | PE-Cy7 | MP4-25D2 | biolegend | ICS TfH |
